# Maintenance of *S*-nitrosothiol homeostasis plays an important role in growth suppression of estrogen receptor-positive breast tumors

**DOI:** 10.1186/bcr3366

**Published:** 2012-12-05

**Authors:** Amanda Cañas, Laura M López-Sánchez, Araceli Valverde-Estepa, Vanessa Hernández, Elena Fuentes, Juan R Muñoz-Castañeda, Chary López-Pedrera, Juan R De La Haba-Rodríguez, Enrique Aranda, Antonio Rodríguez-Ariza

**Affiliations:** 1 Oncology Department, Instituto Maimónides de Investigación Biomédica de Córdoba (IMIBIC), Hospital Reina Sofía, Avda. Menendez Pidal s/n, Córdoba 14004, Spain; 2 Research Unit, Instituto Maimónides de Investigación Biomédica de Córdoba (IMIBIC), Hospital Reina Sofía, Avda. Menendez Pidal s/n, Córdoba 14004, Spain; 3 Cell Biology Department, Instituto Maimónides de Investigación Biomédica de Córdoba (IMIBIC), Universidad de Córdoba, Avda. Menendez Pidal s/n, Córdoba 14004, Spain; 4 Pathology Department, Hospital Reina Sofía, Avda. Menendez Pidal s/n, Córdoba 14004, Spain

## Abstract

**Introduction:**

Protein denitrosylation by thioredoxin reductase (TrxR) is key for maintaining *S*-nitrosothiol (SNO) homeostasis, although its role in tumor progression is unknown. Therefore, the present study aimed to assess the role of altered SNO homeostasis in breast cancer cells.

**Methods:**

The impairment of SNO homeostasis in breast cancer cells was achieved with the highly specific TrxR inhibitor auranofin and/or exposure to *S*-nitroso-L-cysteine. *S*-nitrosylated proteins were detected using the biotin switch assay. Estrogen receptor (ER) alpha knockdown was achieved using RNA silencing technologies and subcellular localization of ERα was analyzed by confocal microscopy. The Oncomine database was explored for TrxR1 (TXNRD1) expression in breast tumors and TrxR1, ER and p53 expression was analyzed by immunohistochemistry in a panel of breast tumors.

**Results:**

The impairment of SNO homeostasis enhanced cell proliferation and survival of ER+ MCF-7 cells, but not of MDA-MB-231 (ER-, mut p53) or BT-474 (ER+, mut p53) cells. This enhanced cell growth and survival was associated with Akt, Erk1/2 phosphorylation, and augmented cyclin D_1 _expression and was abolished by the ER antagonist fulvestrant or the p53 specific inhibitor pifithrin-α. The specific silencing of ERα expression in MCF-7 cells also abrogated the growth effect of TrxR inhibition. Estrogenic deprivation in MCF-7 cells potentiated the pro-proliferative effect of impaired SNO homeostasis. Moreover, the subcellular distribution of ERα was altered, with a predominant nuclear localization associated with phosphorylation at Thr311 in those cells with impaired SNO homeostasis. The impairment of SNO homeostasis also expanded a cancer stem cell-like subpopulation in MCF-7 cells, as indicated by the increase of percentage of CD44^+ ^cells and the augmented capability to form mammospheres *in vitro*. Notably, ER+ status in breast tumors was significantly associated with lower TXNDR1 mRNA expression and immunohistochemical studies confirmed this association, particularly when p53 abnormalities were absent.

**Conclusion:**

The ER status in breast cancer may dictate tumor response to different nitrosative environments. Impairment of SNO homeostasis confers survival advantages to ER+ breast tumors, and these molecular mechanisms may also participate in the development of resistance against hormonal therapies that arise in this type of mammary tumors.

## Introduction

One key mechanism by which nitric oxide (NO) regulates the function of target proteins is through the coupling of a nitroso moiety to a reactive thiol group in specific cysteine residues, leading to the formation of *S*-nitrosothiol (SNO), a process usually known as *S*-nitrosylation [1-3]. Recent research has uncovered the key role of enzyme-mediated processes in the nitrosylation and denitrosylation of proteins and therefore in the maintenance of SNO homeostasis [4,5]. The thioredoxin/thioredoxin reductase (Trx/TrxR) system is one of the specific enzymatic systems regulating basal and stimulus-induced protein denitrosylation. The Trx/TrxR system denitrosylates low molecular weight SNOs as well as SNO proteins [6,7], and we and others have shown that increased protein nitrosylation levels are observed when cells are treated with auranofin, a highly specific TrxR inhibitor [7-9].

A broad spectrum of pathologies, including cardiovascular diseases [10], respiratory diseases [11,12], hepatic diseases [13], neurodegenerative diseases [14,15] and neoplasic diseases [16], has been associated with impaired SNO homeostasis and aberrant *S*-nitrosylation of proteins. In this regard, we have recently reported that the inhibition of NO synthesis during induced cholestasis ameliorates hepatocellular injury, and that this therapeutic effect is in part mediated by the improvement of liver proficiency in maintaining SNO homeostasis [13].

Evidence is accumulating that *S*-nitrosylation has a key role in cancer [17] and, given the importance of SNO homeostasis in the proper regulation of this post-translational modification, this aspect must also be taken into account when investigating tumor biology. However, although the participation of NO in the process of tumorigenesis and tumor progression is well recognized, there are scarce studies addressing the participation of SNO metabolism in cancer. The present study aimed to assess the role of the alteration of SNO homeostasis in breast cancer cells. Unexpectedly, we found that impairment of SNO homeostasis may confer survival advantages to estrogen receptor (ER)-positive breast cancer cells. Indeed, positive ER status in breast tumors was found to be associated with significantly lower levels of TrxR/TXNDR1 expression compared with ER- tumors. Our results suggest that the ER status in breast cancer may dictate the tumor response to impaired SNO homeostasis, and the molecular mechanisms involved may also play a significant role in the development of resistance against hormonal therapies.

## Materials and methods

The experimental research complies with the institution's guidelines and was conducted with the approval of the Ethics Committee from the Reina Sofia Hospital, Cordoba, Spain. Patient consent was not required for this study because the de-identified human breast tumor samples were from a paraffin tissue array of breast tumors provided by Biochain (Newark, CA, USA).

### Materials

Auranofin (1-thio-β-D-glucopyranosatotriethyl phosphine gold-2,3,4,6-tetraacetate) was obtained from Enzo Life Sciences (Exeter, UK). Fulvestrant (ICI 182,780), pifithrin-α and 17β-estradiol were from Sigma-Aldrich (Madrid, Spain). Stock solutions of auranofin or 17β-estradiol (prepared in ethanol) and of fulvestrant or pifithrin-α (prepared in dimethylsulfoxide) were diluted in culture medium and added to cells as described in figure legends. The final concentration of vehicle (ethanol or dimethylsulfoxide) was 0.01%. *S*-nitrosocysteine (CSNO) was synthesized as described elsewhere [18] by incubation of L-cysteine with acidified sodium nitrite and quantification by absorbance at 334 nm using a molar absorption coefficient of 0.74/mM/cm.

### Cell culture

MCF-7, MDA-MB-231 and BT-474 cells were from the European Collection of Cell Cultures (ECACC, Salisbury, UK) and obtained through Sigma-Aldrich. Cells were grown in MEM with Earle's salts (PAA Laboratories GmbH, Pasching, Austria), containing 15% fetal bovine serum (PAA Laboratories) and supplemented with 2 mM glutamine, 1% non-essential amino acids, penicillin (100 U/ml), streptomycin (100 μg/ml) and amphotericin B (2.5 μg/ml).

### Cell proliferation and cell death assays

Cells were seeded in 96-well plates and treated as described in the figure legends for 3 days with a medium change on day 2. After 48 or 72 hours of treatment, cell proliferation was assayed using the XTT Cell Proliferation Assay Kit (Roche, Basel. Switzerland) following the manufacturer's instructions. The formazan dye produced through the reduction of XTT tetrazolium salt by dehydrogenases and reductases of viable cells was detected espectrophotometrically (450 to 655 nm) using a Imark™ Microplate Reader (Biorad, Hercules, CA, USA). In each assay, cell proliferation was expressed as the percentage of untreated cells. Apoptotic cell death was measured using fluorescein isothiocyanate-conjugated annexin V/propidium iodide assay (Bender MedSystems Inc., Vienna, Austria) following the manufacturer's recommendations. Flow cytometry was performed in a FACSCalibur (BD Biosciences, San Jose, CA, USA) to quantify the percentage of apoptotic cells.

### Immunoblotting

Cells grown in 60 mm dishes were harvested with cold PBS, and after centrifugation (1,500 × *g*, 4°C, 5 minutes) the cell pellet was incubated for 15 minutes on ice with 1 ml lysis buffer (50 mM Tris-HCl (pH 7.4), 150 mM NaCl, 5 mM ethylenediamine tetraacetic acid (EDTA), 1 mM ethyleneglycol tetraacetic acid (EGTA), 1.5 mM MgCl_2_, 10% glycerol, 1% NP40, 0.1 M dithiothreitol (DTT), 0.1 M phenylmethylsulfonyl fluoride (PMSF), 1% v/v protease inhibitor cocktail (SERVA, Heidelberg, Germany) and 1% v/v phosphatase inhibitor cocktails 2 and 3 (Sigma-Aldrich)) and centrifuged at 15,000 × *g *for 15 minutes at 4°C. Cell lysates were stored at 80°C until analysis. To obtain cytosolic and nuclear fractions, cells were incubated for 15 minutes at 4°C in low ionic strength lysis buffer A (10 mM HEPES (pH 7.9), 10 mM KCl, 0.1 mM EDTA, 0.1 mM EGTA, 0.1 M DTT, 0.1 M PMSF, 1% v/v protease inhibitor cocktail and 1% v/v phosphatase inhibitor cocktails), vortexed for 10 seconds and centrifuged at 14,000 × *g *for 5 minutes at 4°C, and supernatants (cytosol fractions) were collected and stored at -80°C until use. The remaining nuclear pellets were incubated on ice in high ionic strength lysis buffer B (20 mM HEPES (pH 7.9), 0.4 M NaCl, 1 mM EDTA, 1 mM EGTA, 0.1 M DTT, 0.1 M PMSF, 1% v/v protease inhibitor cocktail and 1% v/v phosphatase inhibitor cocktails) for 15 minutes, with repeated vortexing, and centrifuged at 14,000 × *g *for 5 minutes at 4°C, and then supernatants (nuclear fractions) were collected and stored at -80°C until use.

The total protein content of the lysates was determined by a standard Bradford assay using the reagent from Bio-Rad Laboratories (Hercules, CA, USA). Proteins were separated on SDS polyacrylamide gel and transferred to a nitrocellulose membrane. The membrane was probed with primary antibodies dissolved in Tris buffered saline with 0.2% Tween 20 followed by incubation with secondary antibody conjugated with horseradish peroxidase, a chemiluminescent reaction with the ECL Plus Western Blotting Detection System or ECL Advance Western Blotting Detection Kit (GE Healthcare Life Sciences, Little Chalfont, UK) and protein bands were visualized by autoradiography by exposing the membrane to X-ray film. Densitometric analyses of protein bands detected were performed with image-J software (National Institutes of Health, Bethesda, MD, USA).

Sources of antibodies were as follows: monoclonal anti-phospho-Akt (Ser473), polyclonal anti-Akt, monoclonal anti-phospho-ERK1/2 (Thr202/Tyr204), polyclonal anti-ERK1/2, monoclonal anti-cyclin D_1_, and monoclonal anti-ERα were from Cell Signaling (Beverly, MA, USA). Polyclonal anti-p53, polyclonal anti-TFIIB, polyclonal anti-actin and secondary antibodies conjugated with horseradish peroxidase were from Santa Cruz Biotechnology (Santa Cruz, CA, USA).

### Detection of protein *S*-nitrosylation by the biotin switch method

The procedure was performed as previously described [13]. Briefly, cells lysates obtained in lysis solution (50 mM Tris-HCl, pH 7.4, 300 mM NaCl, 5 mM EDTA, 0.1 mM neocuproine, 1% Triton X-100 and 1 mM PMSF plus aprotinin and leupeptin) were incubated with 20 mM methyl methanethiosulfonate (Sigma-Aldrich, Madrid, Spain) followed by acetone precipitation. Precipitates were centrifuged and resuspended in HENS buffer (250 mM HEPES (pH 7.7), 1 mM EDTA, 0. 1 mM neocuproine, and 1% SDS) and then incubated with 1 mM ascorbic acid and 4 mM *N*-(6-(biotinamido)hexyl)-3'-(2'-pyridyldithio)propionamide (biotin-HPDP; Pierce, Rockford, IL, USA) for 1 hour. Because biotin-HPDP is cleavable under the reduced conditions, prepared samples were loaded onto SDS-PAGE gels without DTT. All steps preceding SDS-PAGE were carried out in the dark. Biotinylated samples were then detected by immunoblotting using a primary monoclonal anti-biotin antibody (Sigma-Aldrich).

### Knockdown of ERα with specific siRNA

ESR1 gene-specific siRNA (Hs_ESR1_8 FlexiTube siRNA) and nonspecific siRNA were obtained from QIAGEN (Hilden, Germany) and used according to the manufacturer's instructions. In brief, siRNA was dissolved (600 ng/ml) in MEM medium, 0% fetal bovine serum and incubated with Hyperfect Transfection Reagent (QIAGEN) for 10 minutes at room temperature. Cells were incubated with the transfection complexes under their normal growth conditions for 24 hours, and then the medium was replaced to perform the different treatments in the transfected cells.

### Immunofluorescent confocal microscopy

Cells grown on sterile glass coverslips in six-well plate were fixed in 4% paraformaldehyde in PBS at room temperature for 20 minutes. Cells were permeabilized in 1% BSA and 0.3% Triton X-100 for 1 hour at room temperature. The cells were incubated with Alexa Fluor-488-conjugate anti-ERα (Santa Cruz Biotechnology) and Alexa Fluor-647-conjugate anti-p53 (Cell Signaling) antibodies overnight at 4°C. Cells were washed with PBS and incubated with 4',6-diamidino-2-phenylindole (Invitrogen, Carlsbad, CA, USA) for nuclear staining. Confocal images were captured under Zeiss LSM 5 Exciter confocal microscope using ZEN 2008 software (Carl Zeiss, Jena, Germany), and analyzed with image-J software (National Institutes of Health).

### Analysis of CD24/CD44 expression and mammosphere formation assay

Expression of cell surface markers CD24 and CD44 in MCF-7 cells or mammospheres was examined by flow cytometry. In brief, adherent cells or mammospheres were harvested, trypsinized, washed in PBS and labeled with monoclonal anti-CD24 phycoerythrin and anti-CD44 fluorescein isothiocyanate antibodies (eBioscience, San Diego, CA, USA), using the corresponding IgG isotypes as controls, and analyzed in a FACSCalibur (BD Biosciences). For the mammosphere formation assay, after treatment MCF-7 cells were tripsinized, counted and re-seeded at clonal density (1 cell/μl) in ultra-low attachment plates (Costar, Corning, NY, USA) in Dulbecco's MEM Nutrient Mixture F+12 Ham supplemented with 10 ng/ml basic fibroblast growth factor, 20 ng/ml epidermal growth factor, 5 μg/ml insulin, 4 mg/ml BSA and 0.5% v/v methylcellulose (R&D Systems, Minneapolis, MN, USA) to prevent cell aggregation. The supplements were added every 2 days and the numbers of mammospheres were counted on day 7 after seeding.

### Immunohistochemical analyses

Immunohistochemical analyses were performed in a paraffin tissue array of breast tumors (Biochain, Newark, CA, USA). Paraffin sections (4 μm) on poly-L-lysine-coated slides were used after drying for 30 minutes at 60°C. The sections were dewaxed in xylene, rehydrated in ethanol and incubated at 100°C in ChemMate™ Target Retrieval Solution (Dako, Barcelona, Spain), pH 6.0 (TrxR1) or pH 9.0 (ER, p53), for 20 minutes. After washing in PBS, the sections were incubated for 10 minutes in 3% hydrogen peroxide to block endogenous peroxidase, and then incubated for 30 minutes with monoclonal anti-TrxR1 (Santa Cruz Biotechnology), anti-ER-α clone EP1 and anti-p53 clone DO7 (Dako) for 1 hour at 4°C. After washing for 5 minutes in PBS, the slides were incubated for 30 minutes with a horseradish peroxidase-labeled polymer (DAKO Envision™ System) and developed for 3 minutes using diaminobenzidine. Finally, the slides were counterstained with hematoxylin and mounted in Eukitt mounting medium. Microscopy images were obtained using a Coolscope digital microscope (Nikon, Tokyo, Japan). The ER and p53 status was defined as positive when showing a minimum of 10% nuclear staining for ERα and p53, respectively. TrxR1 expression was semiquantitatively scored on TrxR1 immunostained sections as absent (0), mild (1), moderate (2), or intense (3). Sections were independently scored by two experienced pathologists.

### Statistical analysis

Reported values are the means ± standard error of the mean (*n *= 3) and statistical comparisons were determined with two-tailed Student's *t *tests. Associations between the ER status and clinical characteristics were assessed by chi-square test. All *P *values resulted from two-sided tests and were considered significant when *P *< 0.05. The significance of the difference between medians of data obtained from the Oncomine database was calculated as described by McGill and colleagues [19].

## Results

**Figure 1:**
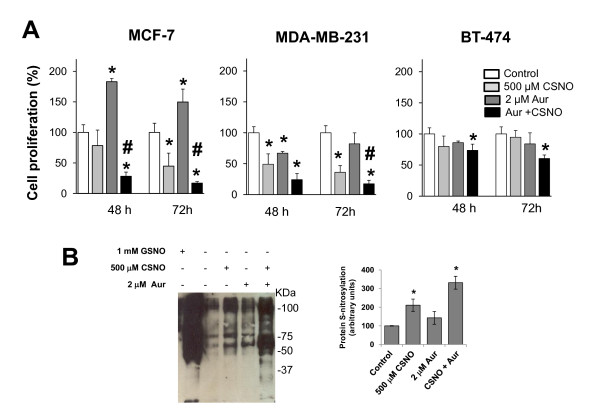
**Severe nitrosative stress reduces cell proliferation in breast cancer cells**. **(A) **Cells were exposed to the indicated treatments and cell proliferation was determined after 48 or 72 hours. Cell proliferation expressed as the percentage of untreated cells. *P < 0.05, compared with untreated. #*P *< 0.05, compared with *S*-nitrosocysteine (CSNO) treated. **(B) **Cell lysates from MCF-7 cells, pretreated or not with auranofin (Aur), and subjected to the indicated treatments were analyzed with the biotin switch assay for the detection of *S*-nitrosylated proteins. The corresponding densitometric analysis of the total protein bands detected in the biotin switch assay (normalized to the signal of Ponceau S stain) is also shown. Data are means ± standard error of the mean of three independent experiments. Significant augmented protein *S*-nitrosylation (*P *< 0.05) was observed comparing CSNO and Aur + CSNO with untreated cells.

To determine the effect that impaired SNO homeostasis may have on cancer cell growth, breast cancer cells were subjected to treatment with the nitrosothiol CSNO in the absence of or in the presence of the TrxR specific inhibitor auranofin. As shown in Figure 1A, treatment with 500 μM CSNO reduced cell proliferation in all three cell lines used, especially when TrxR was inhibited. Consistent with these results, MCF-7 cells treated with 500 μM CSNO exhibited an increase in *S*-nitrosylated proteins, as detected by the biotin switch method (Figure 1B). Moreover, pretreatment with auranofin enhanced the levels of nitrosoproteins in breast cancer cells.

**Figure 2:**
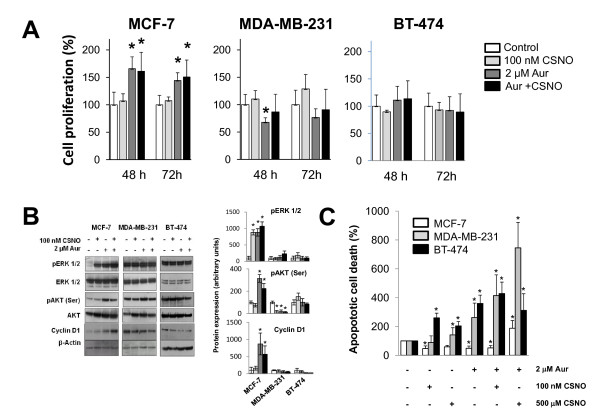
**Mild nitrosative stress increases cell proliferation and survival of MCF-7 cells**. **(A) **Cells were exposed to the indicated treatments and cell proliferation was determined after 48 or 72 hours. Cell proliferation expressed as the percentage of untreated cells. **(B) **Cells were exposed for 6 hours to the indicated treatments and phosphorylation of Akt and Erk1/2 and cyclin D_1 _levels was determined by western blot using the corresponding specific antibodies. The corresponding densitometric analyses of the protein bands detected in the immunoblots and normalized to the signal of β-actin are also shown. Data are means ± standard error of the mean of three independent experiments. **(C) **Cells were exposed to the indicated treatments and apoptotic cell death was determined. The percentage of apoptotic cells in controls was 7.4 ± 1.74, 2.4 ± 0.94 and 2.2 ± 1.04 for MCF-7 cells, MDA-MB-231 cells and BT-474 cells, respectively. Apoptotic cell death expressed as the percentage of untreated cells. *P < 0.05, compared with untreated. #*P *< 0.05, compared with *S*-nitrosocysteine (CSNO) treated. Aur, auranofin.

However, despite the antiproliferative effect obtained by a severe alteration of SNO homeostasis, the inhibition of TrxR with auranofin in MCF-7 cells induced a pro-proliferative effect. Besides, as shown in Figure 2A, the treatment with auranofin in the presence or in the absence of a lower dose of nitrosothiol (100 nM CSNO) increased proliferation in MCF-7 (ER+) cells but not in MDA-MB-231 (ER-, p53 mut) or BT-474 (ER+, p53 mut) cells. Therefore, although a severe nitrosative stress caused growth arrest in breast cancer cells, a mild nitrosative stress promoted the growth of tumor cells in an ER+ and intact p53 setting.

Cellular signaling pathways involved in cell proliferation and survival were analyzed to further explore the mechanisms responsible for the pro-proliferative effect exerted by mild nitrosative stress in breast cancer cells. Analysis by immunoblotting of the phosphorylated forms of ERK1/2 and Akt revealed that 100 nM CSNO, auranofin, or a combination of both treatments increased phosphorylation of ERK1/2 and Akt, but only in MCF-7 cells (Figure 2B). Notably, these treatments also induced in MCF-7 cells the expression of cyclin D_1_, a protein required for cell cycle G_1_/S transition. Thus, in MCF-7 (ER+) cells, mild conditions of nitrosative stress induce the activation of intracellular signaling pathways aimed at cell survival and proliferation. Notably, only in MCF-7 cells, but not in MDA-MB-231 or BT-474 cells, the treatment with auranofin or 100 nM CSNO significantly reduced the rate of apoptosis, confirming the activation of survival pathways in MCF-7 cells. Only when a severe nitrosative stress was induced in MCF-7 cells, which induced massive *S*-nitrosylation of proteins, was an increase in apoptotic death observed (Figure 2C).

**Figure 3:**
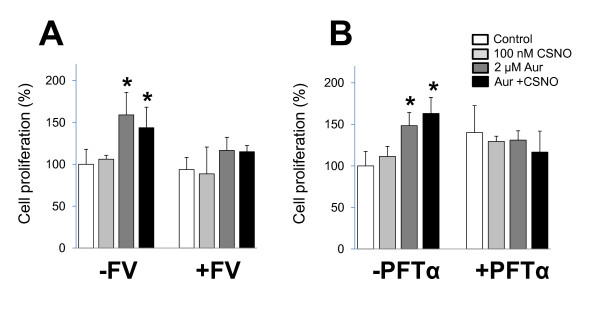
**Proliferative effect of mild nitrosative stress is abolished by ERα antagonist or p53 inhibitor**. MCF-7 cells were preincubated with **(A) **100 nM fulvestrant (FV) or **(B) **10 μM pifitrin-α (PFTα) before the indicated treatments and cell proliferation was determined after 48 hours. Cell proliferation expressed as percentage of untreated cells. *P < 0.05, compared with untreated. ER, estrogen receptor. Aur, auranofin; CSNO, *S*-nitrosocysteine.

The proliferative effect caused by moderate nitrosative stress in breast cancer cells was not observed in cells that do not express ERα (MDA-MB-231), or, even in cells expressing ERα, also carried a mutation in p53 (BT-474). The role of both proteins was therefore explored by treating MCF-7 cells with fulvestrant, an antagonist of ERα, or with pifitrin-α, an inhibitor of p53. As shown in Figure 3, pretreatment of MCF-7 cells with 100 nM fulvestrant or 10 μM pifitrin-α completely abrogated the proliferative effect of moderate nitrosative stress. Functional ERα and active p53 are therefore prerequisites for the cellular proliferation directed by mild conditions of nitrosative stress in breast cancer cells.

**Figure 4:**
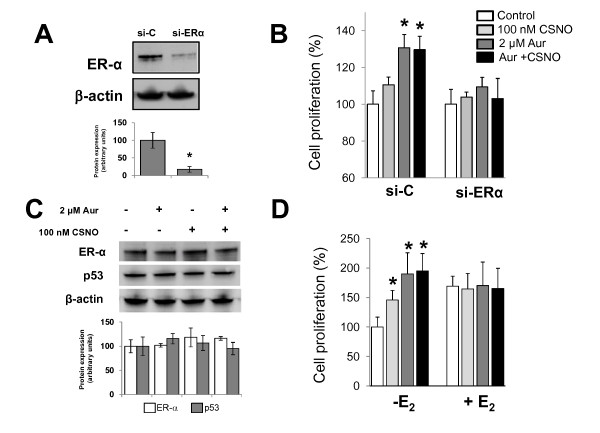
**Proliferative effect of mild nitrosative stress abolished by ERα silencing and increased by estrogen deprivation**. **(A) **Cells were transiently transfected with scrambled siRNA (si-C) or specific si-ERα as described in Materials and methods and ERα expression was analyzed by immunoblotting in whole cell lysates. The corresponding densitometric analysis of the protein band detected and normalized to the signal of β-actin in the immunoblot is also shown. Data are means ± standard error of the mean of three independent experiments. *P < 0.05, compared with cells transfected with si-C. **(B) **Cells were transiently transfected with si-C or si-ERα, treated as indicated, and cell proliferation was determined after 48 hours of treatment. Cell proliferation expressed as the percentage of untreated cells. *P < 0.05, compared with untreated. **(C) **Cells were subjected to the indicated treatments and ERα and p53 expression was analyzed by immunoblotting. The corresponding densitometric analysis of the protein band detected and normalized to the signal of β-actin in the immunoblot is also shown. Data are means ± standard error of the mean of three independent experiments. *P < 0.05, compared with untreated cells. **(D) **MCF-7 cells were maintained in phenol red-free medium with charcoal-stripped serum for 24 hours before exposure to the indicated treatments. Cell proliferation expressed as the percentage of untreated cells. *P < 0.05, compared with untreated. Aur, auranofin; CSNO, *S*-nitrosocysteine; E_2_, 17β-estradiol; ER, estrogen receptor.

To confirm the involvement of ERα in the proliferative effect of auranofin in MCF-7 cells, a series of experiments with a siRNA specific for the ESR1 gene were performed. As shown in Figure 4A, the specific silencing of the ESR1 gene (si-ER-α) in MCF-7 cells significantly reduced the expression of ERα protein. Notably, under these conditions where ERα is not expressed, the proliferative effect of TrxR inhibition in the presence or the absence of 100 nM CSNO was abrogated (Figure 4B). The expression of ERα is thus required for the augmented cell proliferation under moderate conditions of nitrosative stress in breast tumor cells. However, exposure of MCF-7 cells to auranofin, in the presence or in the absence of CSNO, did not significantly alter the levels of ERα or p53 proteins (Figure 4C). The increased proliferative capacity of ER+ breast cancer cells subjected to mild nitrosative stress therefore arises through mechanisms altering the functionality of ERα and/or p53.

The results obtained in the previous experiments suggested that the increase in cell proliferation observed in ER+/p53wt breast cancer cells when exposed to moderate nitrosative stress conditions could be related to a response similar to estrogenic response. Consequently, we conducted a series of experiments in which the MCF-7 cells were deprived of any estrogenic stimulus before exposure to the different treatments. To this end, cells were maintained in phenol red-free medium and charcoal-stripped serum, prior to treatment with auranofin and/or 100 nM CSNO. As shown in Figure 4D, estrogen deprivation further increased the proliferative effect of moderate nitrosative stress in breast cancer cells. Under these conditions, the treatment of cells with 100 nM CSNO increased cell proliferation by 50%; and when cells were treated with auranofin, cell proliferation was increased twofold compared with untreated cells. Significantly, when the cells were previously exposed to 10 nM estradiol, this proliferative effect was not observed (Figure 4D). These experiments therefore indicate that moderate impairment of SNO homeostasis induces a response similar to estrogen and also suggest that mild nitrosative stress may facilitate cell proliferation in breast cancer through an ER-mediated pathway.

**Figure 5:**
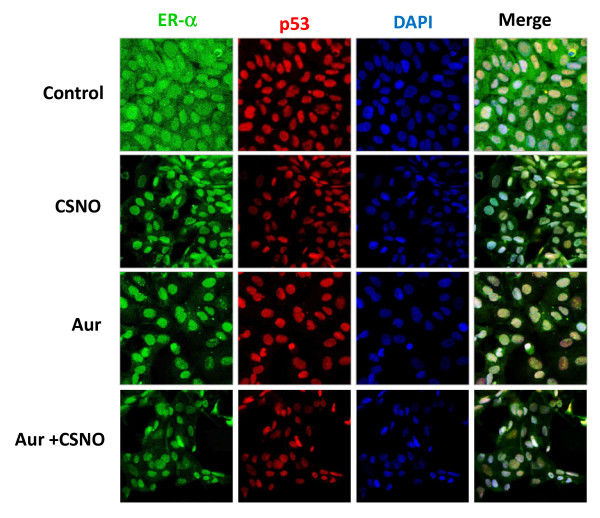
**Subcellular localization of ERα is altered by the impairment of *S*-nitrosothiol homeostasis**. **(A) **To determine subcellular localization of ERα and p53, estrogen-deprived MCF-7 cells were exposed for 6 hours to the indicated treatments and stained for ERα (green) and p53 (red) immunofluorescence and counterstained with 4',6-diamidino-2-phenylindole (DAPI; blue). Merged images of ERα, p53 and DAPI staining are also shown. Original magnification: 40 ×. Aur, auranofin; CSNO, *S*-nitrosocysteine; ER, estrogen receptor.

We next investigated whether the impairment of SNO homeostasis may alter the subcellular distribution of ERα. To this end, estrogen-deprived MCF-7 cells were treated with 100 nM CSNO, auranofin, or both and confocal microscopy was performed to study the subcellular distribution of ERα (Figure 5). Since p53 also appeared to be involved in the observed increase in proliferation, the subcellular distribution of this protein was also analyzed. As shown in Figure 5, treatment of estrogen-deprived MCF-7 cells with CSNO or auranofin, added separately or in combination, induced an almost exclusively nuclear localization of ERα, with a clear reduction of the cytoplasmic levels of the receptor. In estrogen-starved MCF-7 cells, confocal analysis revealed a prevalent nuclear distribution of p53 protein, which was not modified by any of the treatments.

**Figure 6:**
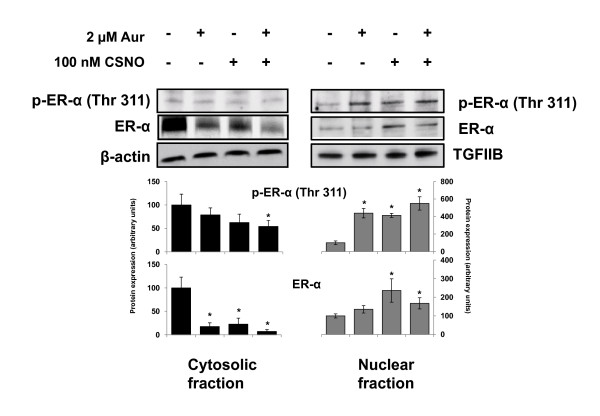
**Alteration of ERα subcellular localization is associated with its phosphorylation at Thr311**. Estrogen-deprived MCF-7 cells were exposed to the indicated treatments for 6 hours and, after separation of cytosolic and nuclear fractions, the expression of ERα and p-ERα (Thr311) was analyzed by immunoblotting. Immunodetection of β-actin and TGFIIB were included as loading controls for cytosolic and nuclear fractions, respectively. The corresponding densitometric analyses of the protein bands detected in the immunoblots and normalized to the signal of β-actin or TFIIB are also shown. Data are means ± standard error of the mean of three independent experiments. *P < 0.05, compared with untreated cells. Aur, auranofin; CSNO, *S*-nitrosocysteine; ER, estrogen receptor; TGF, transforming growth factor.

To confirm the previous results, estrogen-starved MCF-7 cells were exposed to the different treatments and, after separation of cytosolic and nuclear fractions, the expression of ERα was analyzed by immunoblotting. As shown in Figure 6, treatment with auranofin and/or CSNO decreased the levels of cytosolic ERα, compared with control cells, where the absence of the estrogen signal induced higher levels of stabilized cytosolic ERα protein. On the contrary, the impairment of SNO homeostasis increased the presence of this receptor in the nuclear fraction from these cells (Figure 6). Phosphorylation of ERα at Thr311 has been described not only to direct the receptor to the nucleus but also to prevent its return to the cytoplasm due to the alteration of a probable nuclear export sequence [20]. We therefore also explored whether the altered subcellular distribution of ERα by impairment of SNO homeostasis could be associated with phosphorylation of ERα at Thr311. As shown in Figure 6, this phosphorylated form of the receptor was almost undetectable in the cytosolic or nuclear fractions from untreated MCF-7 cells. On the contrary, p-ERα (Thr311) was readily detected in the nuclear fractions from auranofin-treated and/or CSNO-treated breast cancer cells.

**Figure 7:**
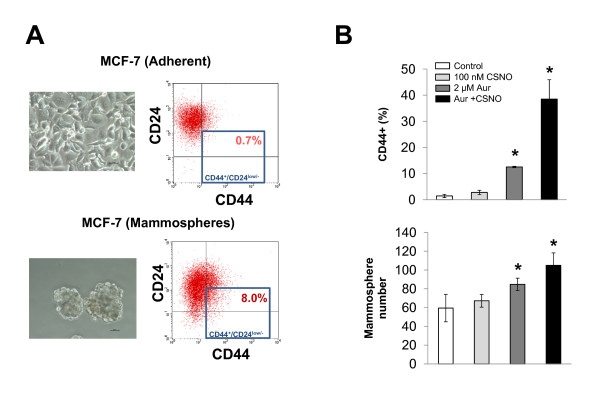
**Impairment of *S*-nitrosothiol homeostasis expands a cancer stem cell-like subpopulation in MCF-7 cells**. (A) MCF-7 cells were grown in suspension in the absence of serum, and the formed mammospheres were then trypsinized, disaggregated and the expression of CD24 and CD44 was analyzed by flow cytometry. The percentage of CD44^+^/CD24^low/- ^cells in mammospheres was compared with that from parental MCF-7 cells. **(B) **Estrogen-starved MCF-7 cells were exposed for 24 hours to the indicated treatments and, after trypsinization and disaggregation, their CD44 expression level was analyzed by flow cytometry and their capability to form mammospheres was evaluated by seeding at clonal density in suspension and in the absence of serum. *P < 0.05, compared with untreated. Aur, auranofin; CSNO, *S*-nitrosocysteine.

Tumors and tumor cell lines constitute a heterogeneous cell population, and only some of these cell subpopulations exhibit increased tumorigenicity and resistance to antitumor therapy. These more aggressive tumor cells are called tumor stem cells or cancer stem cells (CSCs), and appear to be responsible for the processes of tumor progression, recurrence and metastases [21]. Estrogen activity is not only responsible for the normal expansion of the population of stem cells in the mammary epithelium during development but also increases the population of CSCs in the tumoral process [22]. We therefore decided to explore this possibility under our experimental conditions. In the case of breast cancer, identifying those cancer cells with stem cell characteristics can be accomplished by functional assays *in vitro*, such as the mammosphere formation assay. In this assay, cells are grown in suspension in the absence of serum and the surviving cells grow into mammospheres, which are enriched in cancer cells with stem cell characteristics. As shown in Figure 7A, the culture of MCF-7 cells under these conditions resulted in the formation of mammospheres that were then trypsinized, disaggregated and analyzed by flow cytometry showing an enrichment in CD44^+^/CD24^low/- ^cells, a phenotypic characteristic that is considered a marker of CSCs in breast tumors [23-25]. As shown in Figure 7B, flow cytometry analysis indicated an increase in the subpopulation of CD44^+ ^cells when SNO homeostasis was impaired in MCF-7 cells. Moreover, the impairment of SNO homeostasis in MCF-7 cells increased their subsequent capability to form mammospheres when seeded at clonal density. These data therefore indicate that the impairment of SNO homeostasis expands a CSC-like subpopulation in MCF-7 cells.

Taken together, our results indicate that the alteration of SNO homeostasis, either through exposure to nitrosothiols or by the inhibition of TrxR, may constitute an adaptive advantage for ER+ breast tumors. We therefore decided to interrogate the Oncomine database for TXNRD1 expression in breast cancer. From the breast cancer datasets in the database, those with ER status defined for samples and with at least 30 samples in both ER+ and ER- groups were selected for the analysis. As shown in Additional file 1, positive ER status in breast tumors was found to be significantly associated with lower levels of TXNDR1 expression. On the contrary, no significant association was found with HER2 status in the three breast cancer datasets where this condition was defined (Additional file 2). Lower expression of TrxR1/TXNRD1 therefore appears to be linked to the ER-dependent growth of breast cancer.

**Figure 8:**
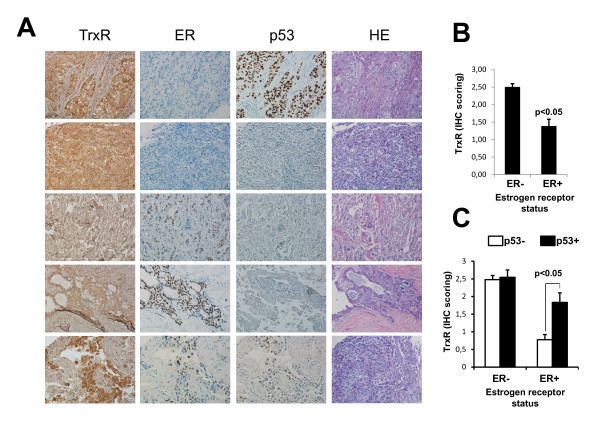
**Positive estrogen receptor status in breast tumors significantly associated with lower levels of TrxR1 expression**. **(A) **TrxR1, estrogen receptor (ER) and p53 expression was analyzed by immunohistochemistry (IHC) in a panel of 57 (36 ER-, 21 ER+) breast tumors. Representative images from IHC and hematoxylin and eosin (HE) staining of ER-/p53+ (row 1), ER-/p53- (row 2), ER+/p53- (rows 3 and 4) and ER+/p53+ (row 5) breast tumors. Original magnification: 20 ×. **(C) **TrxR1 (IHC) scoring in ER- and ER+ tumors. **(D) **TrxR1 (IHC) scoring in p53- and p53+ breast tumors. TrxR, thioredoxin reductase.

To confirm the relationship between the expression of ER and TrxR1 in breast cancer we next analyzed TrxR1, ER and p53 expression by immunohistochemistry in a panel of 57 breast tumors, whose clinical characteristics are summarized in Additional file 3. The specificity of the anti-TrxR1 antibody employed in the immunohistochemistry studies was previously confirmed by western blot and siRNA silencing. The antibody recognized a single 55 kDa protein that was knocked-down by specific silencing of the TXNRD1 gene (Additional file 4). As shown in Figure 8A, ER expression was not observed in some tumors whereas in others ER was immunodetected in the nucleus of tumoral cells, thus defining the ER status of tumors as ER- (*n *= 36) or ER+ (*n *= 21), respectively. Also, p53 expression was evaluated by immunohistochemistry, since p53 accumulation might serve as a surrogate biomarker of p53 mutation in breast carcinomas [26,27]. In our study, 58% of tumors were p53+ as indicated by the immunodetection of p53 protein in the nucleus of tumoral cells (Figure 8A). On the contrary, TrxR1 protein was immunodetected in all of the analyzed breast tumors, mostly in the cytosol of epithelial tumor cells. Importantly, positive ER status in breast tumors was found to be significantly associated with lower levels of TrxR1 expression as detected by immunohistochemistry (Figure 8B). Furthermore, the association of a lower TrxR1 expression with the absence of p53 alterations was observed in ER+ tumors but not in ER- tumors (Figure 8C).

## Discussion

Evidence is accumulating that *S*-nitrosylation has an important role in the NO-mediated regulation of tumorigenesis and tumor progression [17]. However, few mechanisms regarding NO/SNO and cancer have been clearly established. In addition, it has long been evident that cancer is a heterogeneous disease. In this regard it is important to note that breast cancer is not a single disease but is instead a collection of breast diseases that have diverse histopathologies, genetic and genomic variations, and clinical outcomes [28]. Determination of the ER status of the tumor is essential to determine whether or not a breast cancer patient is a candidate for hormonal therapy. Overall, about 50 to 60% of all breast cancers are ER+.

In previous studies we have shown in different experimental models that impairment of the enzymatic systems that maintain SNO homeostasis cause increased cellular *S*-nitrosoprotein levels [9,13]. In the present study the biotin switch assay indicated greater *S*-nitrosylation levels when cells were treated with 500 μM CSNO compared with auranofin. Furthermore, previously we have demonstrated in human hepatocytes that the profile of *S*-nitrosylated proteins after TrxR inhibition with auranofin is different from those identified when cells are treated with CSNO or when *S*-nitrosoglutathione reductase (GSNOR) activity is impaired [9]. Thus, 500 μM CSNO may exceed the capacity of other defense systems participating in nitrosothiol homeostasis, such as GSNOR, and the threshold between mild and nitrosative stress may be determined not only by the level of protein *S*-nitrosylation but also by the type of proteins that are modified.

We have shown that severe nitrosative stress in breast cancer cells, resulting from specific inhibition of one of these enzyme systems regulators and exposure to a high dose of nitrosothiol CSNO, inhibited cell proliferation This growth inhibition was associated with a marked increased level of *S*-nitrosylated proteins detectable by the biotin switch method. The massive *S*-nitrosylation of key proteins for cell survival could explain the inhibitory effect of severe nitrosative stress. These results suggest a potential therapeutic use of drugs capable of altering SNO homeostasis in tumors [9]. However, and significantly, induction of a moderate nitrosative stress was able to stimulate cell proliferation of breast tumor cells in a context of intact ER+/p53. Our results indicate that the effect of impairing SNO homeostasis in cancer cells depends on the intensity of the resulting nitrosative stress and also on the cellular context. This confirms the duality of NO in cancer and underscores the complexity underlying the role that *S*-nitrosylation may play in tumor biology [17].

In the present study, the increased proliferation in ER+ breast cancer cells subjected to a moderate nitrosative stress was associated with the activation of cellular proliferation and survival signaling pathways. Akt, also known as protein kinase B, is a serine/threonine protein kinase that is involved in cell survival pathways by inhibiting apoptotic processes. On the other hand, ERK1/2 are members of the mitogen-activated protein family of kinases that act as key integration points in cell signaling. Both Akt and ERK1/2 are translocated to the cell nucleus upon activation by phosphorylation, and there in turn phosphorylate other molecular targets by inducing the expression of genes involved in proliferation and cell survival. Furthermore, in our hands, activation of these signaling pathways in MCF-7 cells was associated with the induction of cyclin D_1 _expression. This protein is induced early in the G_1 _phase of cell cycle, and the complex formed between cyclin-dependent kinases and cyclin D_1 _is critical for phosphorylation of substrates that are essential for cell proliferation. Significantly, cyclin D_1 _plays an important role in the progression through the cell cycle induced by estrogen and ER [29]. These data therefore suggest that the ERα receptor is involved in the augmented proliferation of MCF-7 cells with impaired SNO homeostasis.

The experiments of ESR1 gene silencing confirmed that the expression of ERα is required for moderate nitrosative stress to increase the proliferative capacity of breast cancer cells. Since the treatments did not alter the levels of expression of this protein, this increase must occur through mechanisms that alter the functionality of ERα. Estrogen deprivation experiments supported this hypothesis, since under these conditions the proliferative effect of SNO homeostasis impairment was enhanced, and this effect was abrogated when cells were previously subjected to an estrogenic signal. Taking together this results indicate that the impairment of SNO homeostasis in ER+ breast cancer cells induces a ER-dependent cell growth response comparable with estrogen response in breast cancer cells. Other studies have shown association between Trx/TrxR and the estrogen response in human breast cancer cells. The impairment of the Trx/TrxR system has thus been described to increase the progesterone receptor expression in MCF-7 cells in response to estrogen [30].

Unlike other nuclear receptors such as thyroid or vitamin D receptors, which reside in the nucleus in the absence of their ligands, estrogenic receptors such as ERα are translocated into the nucleus after ligand binding, whereas pure antagonists such as fulvestrant target ERα to the cytoplasm [20,31]. Our experiments support that fulvestrant disruption of ERα traffic between cytoplasm and nucleus eliminates the pro-proliferative effect of moderate nitrosative stress in breast cancer cells. On the other hand, the intracellular concentration of ERα is the result of a dynamic equilibrium between synthesis and degradation [32]. The absence of an estrogen signal induces higher levels of stabilized cytosolic ER protein, forming a complex with chaperones [33]. This stabilized cytosolic ER pool may explain why the pro-proliferative effects of CSNO and auranofin treatment are enhanced in estrogen-starved cells and abolished by 17β-estradiol. On ligand (17β-estradiol) binding, the receptor dissociates from the complex, and is either ubiquitinated for degradation in the cytoplasm or translocates to the cell nucleus by a nuclear localization signal (domain D), although the molecular mechanisms involved are not well understood [33]. Our data indicate that impairment of SNO homeostasis induces a distinct decrease of cytoplasmic ERα levels, resulting in an almost exclusively nuclear localization of this receptor.

In endometrial tumor cells that express ERα, estradiol induces phosphorylation of ERα at residue Thr311 through activation of the p38 mitogen-activated protein kinase [20]. This phosphorylation not only directs ERα to the nucleus, but also prevents its return to the cytoplasm due to the alteration of a probable nuclear export sequence. Although we did not find p38 activation, p-ERα (Thr311) was readily detected in the nuclear fractions of breast cancer cells when subjected to impairment of SNO homeostasis. Activation of other mitogen-activated protein kinases, such as Akt, due to SNO homeostasis impairment may therefore possibly participate in the phosphorylation of ERα at residue Thr311 and in the alteration of its subcellular distribution in breast cancer cells.

The participation of p53 in the proliferative effect of impaired SNO homeostasis remains uncertain. We have shown that impairment of SNO augments cell growth in MCF-7 cells but not in BT-474 cells, which also express ERα but harbor a mutated p53 protein. Furthermore, a specific inhibitor of p53 also abolished the proliferative effect in MCF-7 cells. Confocal analysis revealed a prevalent nuclear distribution of p53 protein in serum-starved MCF-7 cells, in agreement with previous reports [34]. However, the subcellular localization of p53 was not altered by any of the treatments, hence other p53-related mechanisms might be involved. For example, it is known that ERα is capable of binding to p53 and suppressing its function [35]. In fact, it has been suggested that ERα uses a dual strategy to promote an abnormal cellular proliferation enhancing the transcription of pro-proliferative genes, but also suppressing the transcription of anti-proliferative genes regulated by p53 [36]. Furthermore, normal signaling regulating interactions between ER and p53 have also been suggested to be disrupted in breast CSCs [36]. In our study the impairment of SNO homeostasis in MCF-7 cells expanded a CSC-like subpopulation, and it is conceivable that the disturbances of the interactions between ER and p53 might be involved.

Interestingly, thioredoxin expression has been associated with lower tumor growth in ER+ and p53 intact breast cancers than in cases that had abnormalities in ER or p53 [37]. This study suggested that thioredoxin may play an important role in suppressing the proliferation of ER+/p53 intact breast tumors, and that Trx expression may mean better prognosis in ER+/p53 intact conditions than in other conditions. In our study we found that lower TrxR1 expression is associated with ER+ tumors, particularly when p53 abnormalities are absent, supporting the notion that the Trx/TrxR enzyme system may act as a suppressor of cell proliferation in ER+/p53 intact tumors. Little is known about the possible prognostic value of TrxR expression in breast cancer. However, the association of TXNRD1 expression with HER2 expression and a worse prognosis has been described [38]. Significantly, in that study the authors also found that positive ER status in breast tumors was associated with lower levels of TXNDR1 expression. The potential prognostic value of TXNDR1/TrxR1 in breast cancer may therefore strongly depend on hormonal receptor status of the tumor. Our data are consistent with these studies and provide a functional basis for the association of the alteration of the Trx/TrxR enzymatic system, the impairment of SNO homeostasis and tumor proliferation in an ER+/p53 intact setting. Besides, the majority of breast tumors that develop hormone independence and resistance to endocrine therapies do so despite the continued expression of ERα. Several mechanisms contributing to the progression of breast cancer to hormone independence, including ligand-independent activation of ERα, have been suggested [39]. The molecular mechanisms described in our study may therefore also play a role in the development of resistance against hormonal therapies in breast cancer.

## Conclusions

Our study highlights the importance of SNO metabolism in cancer. The adequate maintenance of SNO homeostasis plays an important role in the suppression of growth of ER+ breast tumors, since the impairment of SNO homeostasis confers survival advantages to ER+ breast cancer cells. Our findings have potential clinical relevance, as they predict that the expression of TrxR or other factors involved in SNO homeostasis could help in the prognosis of ER+ tumors. Also, they provide evidence of other molecular mechanisms contributing to the progression of breast cancers to hormone independence and could facilitate the development of therapeutic options to counteract their eventual hormone independence.

## Abbreviations

Akt: protein kinase B; BSA: bovine serum albumin; CSC: cancer stem cell; CSNO: *S*-nitrosocysteine; DTT: dithiothreitol; EDTA: ethylenediamine tetraacetic acid; EGTA: ethyleneglycol tetraacetic acid; ERK1/2: extracellular signal-regulated kinases 1 and 2; ER: estrogen receptor; MEM: modified Eagle's medium; NO: nitric oxide; PBS: phosphate-buffered saline; PMSF: phenylmethylsulfonyl fluoride; siRNA: small interfering RNA; SNO: *S*-nitrosothiol; Trx: thioredoxin; TrxR: thioredoxin reductase.

## Competing interests

The authors declare that they have no competing interests.

## Authors' contributions

AC, LML-S, AV-E, and VH performed all the experiments and analyses except where noted below. EF and JRM-C performed immunohistochemical analyses. EF, JRDLH-R and EA evaluated the clinical pathological parameters. CL-P, JRM-C, JRDLH-R and EA provided valuable feedback during the project and revised the manuscript draft. AR-A conceived the study, participated in its design and coordination and drafted the manuscript. All authors contributed to the interpretation and discussion of the findings and approved the final manuscript.

## Supplementary Material

Additional file 1 **Association of ER status and TXNRD1 expression in breast cancer**. Eight breast cancer datasets from the Oncomine database with ER status defined for samples and with at least 30 samples in both ER- and ER+ groups were analyzed to study association of ER status and TXNRD1 expression. Click here for file

Additional file 2 **Association of HER2 status and TXNRD1 expression in breast cancer**. Three breast cancer datasets from the Oncomine database with ER and HER2 status defined for samples and with at least 30 samples in both ER- and ER+ groups were analyzed to study association of HER2 status and TXNRD1 expression. Click here for file

Additional file 3 **Pathological characteristics of breast tumors**. Table summarizing the clinical characteristic of the breast tumor panel used. Click here for file

Additional file 4 **Validation of the anti-TrxR1 antibody employed in the immunohistochemical studies**. MCF-7 cells were transiently transfected with scrambled siRNA (si-C) or with two specific si-TrxR1 siRNAs, and TrxR1 expression was analyzed by immunoblotting in whole cell lysates. Click here for file
