# Supplementary material for: Maintenance of S-nitrosothiol homeostasis plays an important role in growth suppression of estrogen receptor-positive breast tumors
Source: Breast Cancer Res. 2012 Dec 5;14(6):R153. doi: 10.1186/bcr3366 (PMC4053140; doi:10.1186/bcr3366)
Supplement: Additional file 1 — Association of ER status and TXNRD1 expression in breast cancer. Eight breast cancer datasets from the Oncomine database with ER status defined for samples and with at least 30 samples in both ER- and ER+ groups were analyzed to study association of ER status and TXNRD1 expression. [file bcr3366-S1.PPT]

## Slide 1
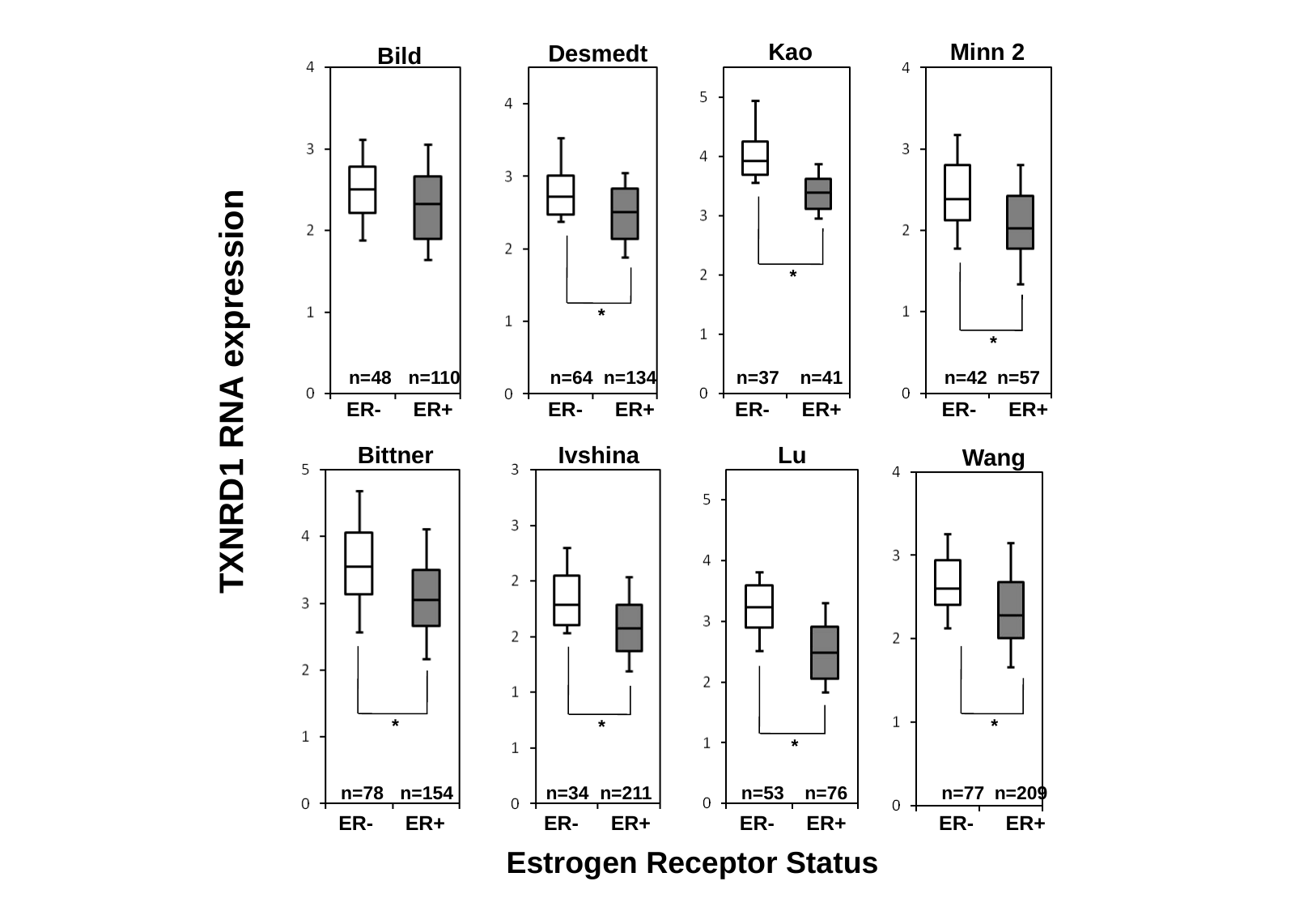

Kao
Minn 2
Desmedt
Bild
*
*
*
n=48
n=110
n=64
n=134
n=37
n=41
n=42
n=57
TXNRD1 RNA expression
ER-
ER+
ER-
ER+
ER-
ER+
ER-
ER+
Bittner
Ivshina
Lu
Wang
*
*
*
*
n=78
n=154
n=34
n=211
n=53
n=76
n=77
n=209
ER-
ER+
ER-
ER+
ER-
ER+
ER-
ER+
Estrogen Receptor Status
