# Supplementary figures and images for: Maintenance of S-nitrosothiol homeostasis plays an important role in growth suppression of estrogen receptor-positive breast tumors
Source: Breast Cancer Res. 2012 Dec 5;14(6):R153. doi: 10.1186/bcr3366 (PMC4053140; doi:10.1186/bcr3366)

## Slide 1
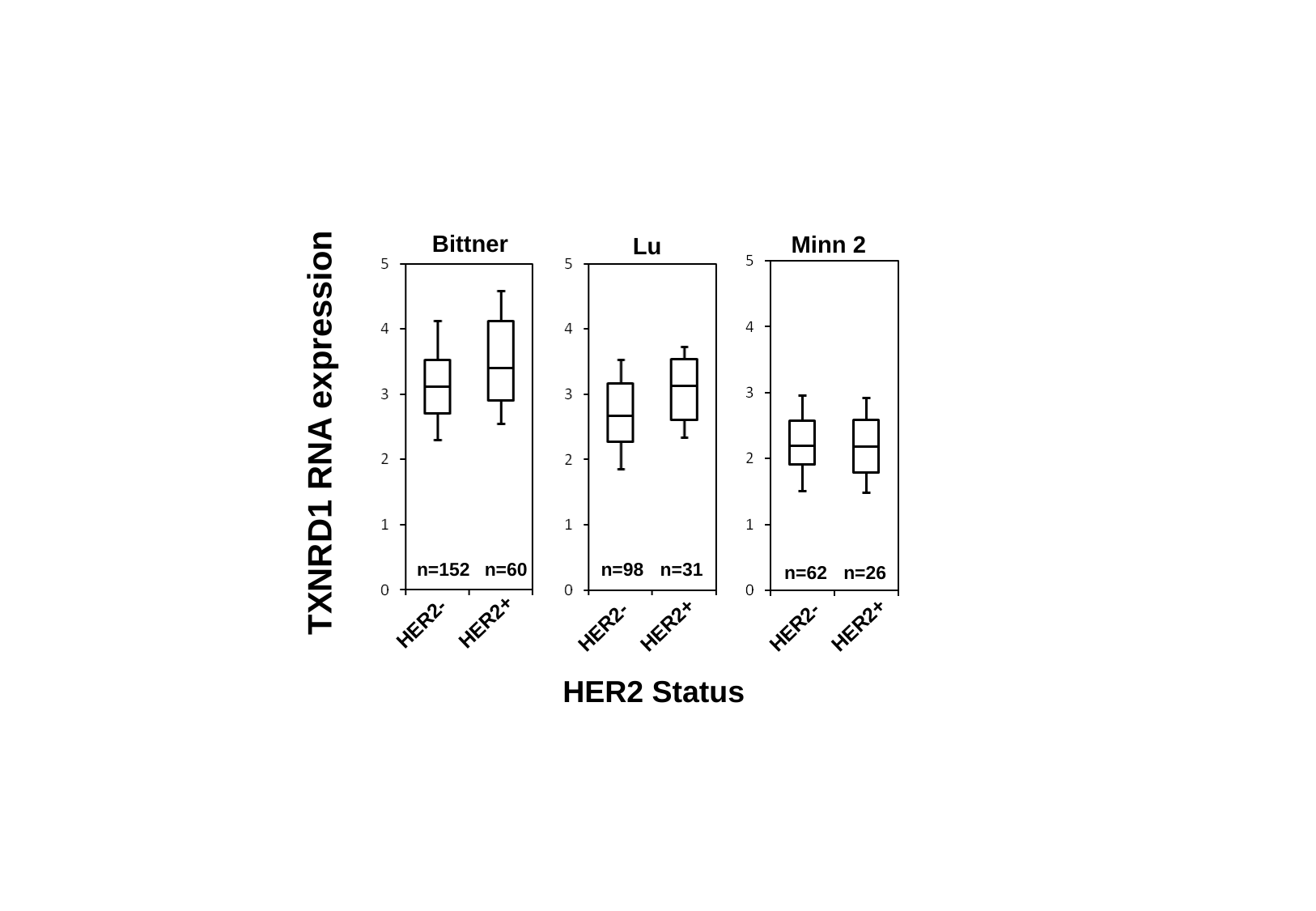

Bittner
Minn 2
Lu
TXNRD1 RNA expression
n=152
n=60
n=98
n=31
n=62
n=26
HER2+
HER2-
HER2+
HER2+
HER2-
HER2-
HER2 Status

Supplement: Additional file 2 — Association of HER2 status and TXNRD1 expression in breast cancer. Three breast cancer datasets from the Oncomine database with ER and HER2 status defined for samples and with at least 30 samples in both ER- and ER+ groups were analyzed to study association of HER2 status and TXNRD1 expression. [file bcr3366-S2.PPT]

## Slide 1
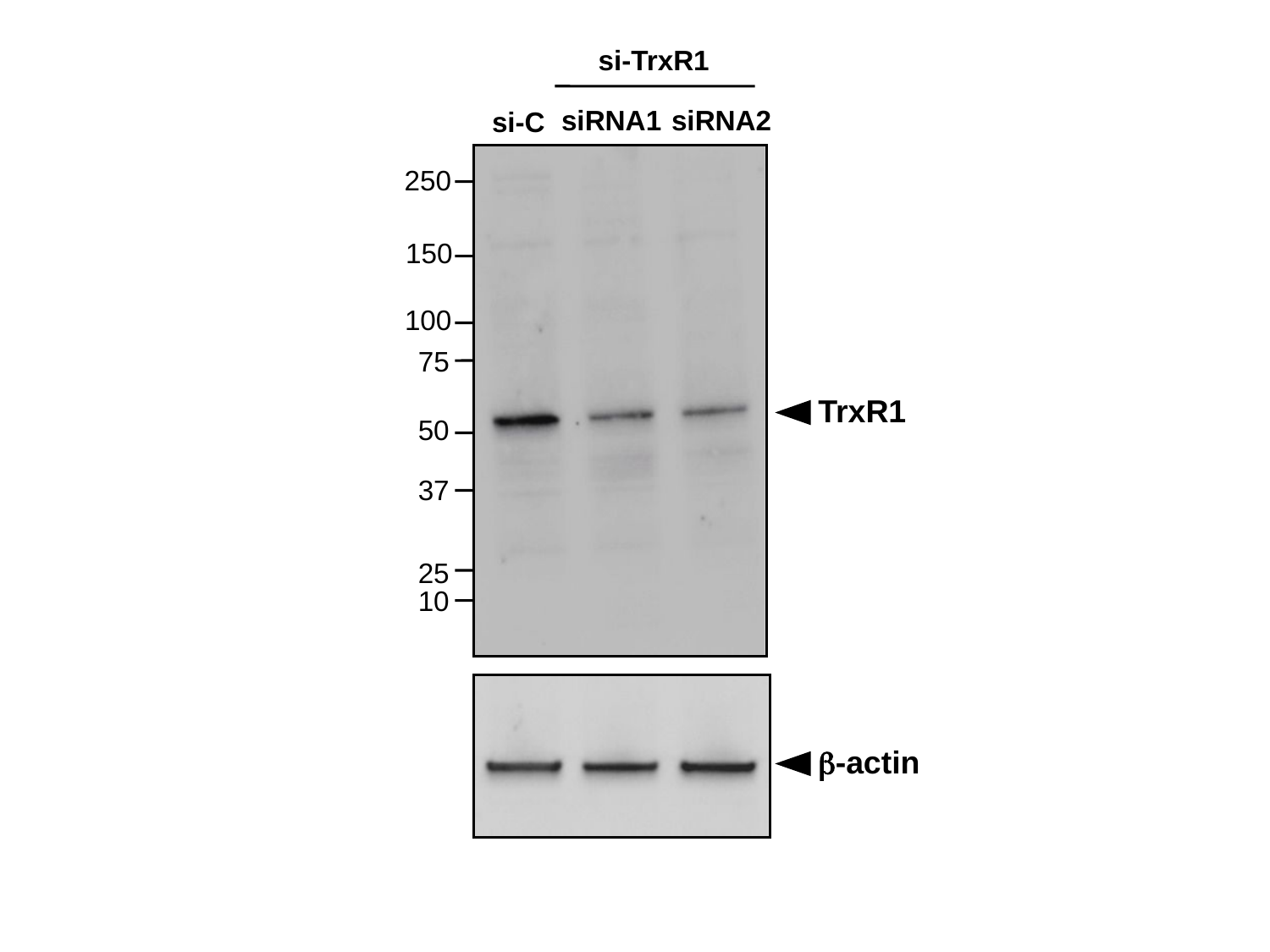

si-TrxR1
siRNA1
siRNA2
si-C
250
150
100
75
TrxR1
50
37
25
10
β-actin

Supplement: Additional file 4 — Validation of the anti-TrxR1 antibody employed in the immunohistochemical studies. MCF-7 cells were transiently transfected with scrambled siRNA (si-C) or with two specific si-TrxR1 siRNAs, and TrxR1 expression was analyzed by immunoblotting in whole cell lysates. [file bcr3366-S4.PPT]
