# Supplementary material for: Maintenance of S-nitrosothiol homeostasis plays an important role in growth suppression of estrogen receptor-positive breast tumors
Source: Breast Cancer Res. 2012 Dec 5;14(6):R153. doi: 10.1186/bcr3366 (PMC4053140; doi:10.1186/bcr3366)
Supplement: Additional file 3 — Pathological characteristics of breast tumors. Table summarizing the clinical characteristic of the breast tumor panel used. [file bcr3366-S3.DOC]

**Supplementary Table1.- Pathological characteristics of breast tumors**

| Characteristics | ER- (n=36) | ER+ (n=21) | *P* value |
| --- | --- | --- | --- |
| Histology | 36 | 21 | 0.101 |
| Invasive ductal | 35 (97.2) | 18 (85.7) |  |
| Invasive lobular | 1 (2.3) | 3 (14.3) |  |
| Tumor size | 35 | 20 | 0.687 |
| T1 | 1 (2.8) | 1 (5.0) |  |
| T2 | 31 (88.6) | 16 (80.0) |  |
| T3 | 3 (8.6) | 3 (15.0) |  |
| T4 | 0 | 0 |  |
| Nodal status | 35 | 20 | 0.438 |
| N0 | 21 (60.0) | 9 (45.0) |  |
| N1 | 7 (20.0) | 4 (20.0) |  |
| N ≥2 | 7 (20.0) | 7 (35.0) |  |
| Metastasis | 35 | 20 | 0.483 |
| M0 | 25 (71.4) | 9 (45.0) |  |
| M1 | 10 (28.6) | 12 (34.3) |  |
| Stage | 35 | 20 | 0.386 |
| I | 1 | 1 |  |
| II | 22 | 11 |  |
| III | 2 | 4 |  |
| IV | 10 | 4 |  |

Pathological details of the breast tumors analyzed were compared according to their ER status. All parameters were well matched between the two study groups. n = number of cases.
